# DNA methylation alterations in the genome of a toddler with cri‐du‐chat syndrome

**DOI:** 10.1002/ccr3.1274

**Published:** 2017-11-20

**Authors:** Oxana Y. Naumova, Sergey Y. Rychkov, Tatyana V. Kuznetzova, Veronika V. Odintsova, Sergey A. Kornilov, Elena L. Grigorenko

**Affiliations:** ^1^ Texas Institute for Measurement, Evaluation, and Statistics University of Houston Houston Texas; ^2^ Laboratory of Human Genetics Vavilov Institute of General Genetics RAS Moscow Russian Federation; ^3^ D.O. Ott's Research Institute of Obstetrics, Gynecology and Reproductology Saint Petersburg Russian Federation; ^4^ Department of Psychology Saint‐Petersburg State University Saint Petersburg Russian Federation

**Keywords:** case study, cri‐du‐chat syndrome, DNA methylation, Illumina Infinium Human Methylation450

## Abstract

This manuscript reports on genomewide epigenetic alterations in cri‐du‐chat syndrome related to a partial aneusomy of chromosome 5. A systematic analysis of these alterations will open up new possibilities for the prognostic evaluation of CDCS patients and the development of new therapeutic interventions for reducing the severity of the disease.

Recent studies have provided evidence supporting the theory that clinical phenotypes related to chromosomal aneuploidies might be due to both the direct involvement of the genes in a damaged genomic region and their indirect effects due to the engagement of these genes in a wide‐ranging cascade of regulatory events that may result in a global remodeling of genomic function. For example, a destabilization of the genome activity has been found in Down syndrome caused by trisomy 21 due to genomewide hypermethylation and significant alterations in the DNA methylation pattern of genes involved in the regulation of chromatin structure 1, 2, 3. Aberrant DNA methylation has been observed in blastocysts with varying autosomal aneuploidies 4, and in patients with Turner and Klinefelter syndromes related to aneuploidies of chromosome X 5.

One of the most common syndromes related to a partial aneusomy of chromosome 5, with an incidence 1:50,000 of live‐born infants, is cri‐du‐chat syndrome (CDCS; OMIM # 123450) 6. A characteristic feature of CDCS is a high‐pitched cat‐like cry in newborns 7. In most cases, CDCS manifests with microcephaly, facial dysmorphology, and severe psychomotor and mental retardation. The size of the deletion in CDCS can vary from extremely small (5p15.2) to the entire short arm of chromosome 5; the majority of deletions arise as de novo mutations. Two of the most critical chromosomal regions related to the cat‐like cry and other clinical features of CDCS have been mapped within 5p15.3 and 5p15.2, respectively 7, 8. The loss of copies of the genes *SEMA5A* and *CTNND2* (5p15.2), which play important roles in cerebral development, is considered to be the primary cause of mental retardation in CDCS 9, 10. To note, the region often found to be critical in the manifestation of CDCS contains a number of genes controlling chromatin activity, such as *PRKAA1*,* BRD9*,* AHRR*, and *MED10* (5p15.3), as well as the methionine synthase reductase gene (*MTRR*; 5p15.31) implicated in the folate metabolism, which provides methyl groups to a variety of cellular pathways, including DNA methylation 11. Recent studies have reported that *MTRR* may affect the methylation of hundreds of loci throughout the genome 12 and might be associated with methylation levels in autoimmune thyroid disease 13. Thus, it is possible that the partial monosomy 5p may lead to aberrations in DNA methylation patterns in CDCS that, in turn, may be related to the development of the CDCS phenotype.

Here, we report on DNA methylation alterations that were observed in the genome of a toddler with CDCS. The participant was the second child (girl) of a Russian woman (37 years of age at the time of this child's birth), born after a 40‐week pregnancy. There were no complications during the pregnancy; the delivery was also normal. The Apgar score was 8 at 5 min after birth; weight and height at birth were 2850 g and 49 cm, respectively, and the chest and head circumferences were 31.5 and 31.5 cm, respectively. During the newborn examination, multiple congenital anomalies were found, including facial dysmorphology, hypertelorism, hypoplasia of the larynx, laryngeal stridor, and microcephaly. This girl was recruited into this study at the age of 31 months. Her medical records were analyzed, a physical examination was conducted, and a blood sample was collected. CDCS was confirmed by the cytogenetic analysis; the girl had the karyotype 46,XX,del(5)(pter‐>p13) (Fig. 1A). Her physical and developmental evaluations registered a number of concomitant conditions, such as a deficiency in bone mass, mental retardation, extrapyramidal insufficiency, congenital myopia, exotropia, partial atrophy of the optic nerves, and pigmentary nevus of the iris; echocardiography revealed patent arterial duct (also known as patent ductus arteriosus – a condition in which a structure called the ductus arteriosus, normal during the fetal period, remains into infancy and onwards, when it should have disappeared). To compare the DNA methylation profile of the patient with CDCS to those of typically developing children, we collected blood from seven girls of similar age (30.79 ± 2.94 mos), who had neither genomic lesions nor any congenital disorder or developmental impairment. This study was approved by the Saint Petersburg University Research Ethics Committee and was performed after obtaining written informed consent from the toddlers’ primary caregivers.

**Figure 1:**
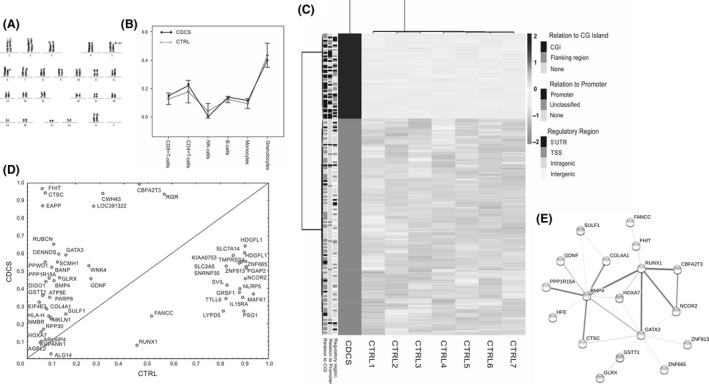
Cytogenetic and epigenetic analysis of the participant with cri‐du‐chat syndrome (CDCS). (A) Karyogram of the girl with CDCS using G‐banding. The deletion of the short arm of chromosome 5 (p13) is shown by the arrow. (B) Plot depicts the variability of blood cell composition in control samples (CTRL; mean ± SD) and in the CDCS sample; the cell count was estimated based on DNA methylation data using the algorithm 15. The plot shows that the distribution of cell types in CDCS lies within the range of individual variability in blood cell types in CTRLs. (C) Heatmap with hierarchical clustering of DNA methylation levels of 191 DMPs (rows) among the CDCS and CTRL samples (columns). Clustering used average linkage and Manhattan distance. Left legend shows the relation of a DMP to regulatory genomic regions: CpG island (CGI), gene promoter, 5′UTR, and 1500 nt upstream of the TSS. (D) Scatterplot represents the *β*‐values of 54 differentially methylated genes in CDCS (*y*‐axis) and controls (*x*‐axis). Points below the diagonal represent genes with lower methylation values and points above the diagonal represent genes with higher methylation values in CDCS. (E) Protein–protein interaction (PPI) network for 54 genes differentially methylated in CDCS visualized by STRING (http://string-db.org/). Only connected nodes (*N* = 18) are shown. The color saturation of the edges represents the confidence score of a functional association. The network has significantly more PPIs (*N* = 24) than expected (*N* = 12); PPI enrichment *P*‐value = 0.004. The network was enriched in such gene ontology terms as the regulation of transcription from an RNA polymerase II promoter (*BMP4*,* CBFA2T3*,* GATA3*,* GDNF*,* HOXA7*,* NCOR2*,* RUNX1*,* PPP1R15A*, and *ZNF665*; enrichment *P*‐value = 0.014), the regulation of embryonic development and organ formation (*BMP4*,* GATA3*,* GDNF*, and *SULF1*;* P* < 0.01), and the regulation of cellular response to growth factor stimulus (*BMP4*,* GATA3*,* HFE*,* NCOR2*,* RUNX1*, and *PPP1R15A*;* P* < 0.01).

We performed genomewide DNA methylation profiling on eight blood samples (the CDCS sample and seven controls, CTRL) using the Illumina Infinium HumanMethylation450 array (Illumina, San Diego, CA, USA). For the data normalization, data processing and differential methylation analysis, the R *minfi* package was applied 14. The probes that showed a significant (> 1.2 fold change at a *P*‐value < 0.05) difference in the methylation level (*β*‐value) in CDCS versus CTRL were defined as differentially methylated probes (DMPs).

First, given the cell specificity of DNA methylation that could bias the differential methylation analysis results, we estimated the intra‐individual variability of blood cell‐type composition using an algorithm for cell count from the DNA methylation data 15. We did not find any significant differences in the cell composition of CDCS in comparison with those in CTRL (Fig. 1B). We identified 191 DMPs that were widespread throughout the genome (Table S1). Most of these DMPs (137 of 191 or 72%) were hypomethylated in CDCS (Fig. 1C), which was expected based on our assumption concerning the insufficiency of the active form of folate – the main donor of methyl groups – due to the deletion involved with *MTRR*. It is necessary to note that these hypomethylation events were found primarily outside of a regulatory region, while the regulatory regions – gene promoters, 5′UTRs, and the regions 1500 nt upstream of a TSS (transcription start site) – were significantly enriched (OR = 26.6, 2.4 and 2.1, respectively, at a *P* < 0.05) with hypermethylation events in CDCS (Fig. 1C).

Second, we focused on genes that exhibited altered methylation within a regulatory element in CDCS, on their functions, and on their associations with human disorders and human abnormal phenotypes as reported in public databases, such as the Gene Ontology resource (http://www.geneontology.org/); the OMIM (Online Mendelian Inheritance in Man; https://omim.org/); and the HPO (The Human Phenotype Ontology; http://human-phenotype-ontology.github.io). We found 54 such differentially methylated genes (DMEG) in CDCS (Fig. 1D; Table S2). Of those 54, 18 DMEGs had known protein–protein interactions (PPI); their PPI network was enriched in functions such as transcriptional regulation, cellular response to growth factor stimulus, and the regulation of embryonic development and organ formation (Fig. 1E). Genes assigned to the last group – *BMP4*,* GATA3*,* GDNF,* and *SULF1*, were hypermethylated and presumably downregulated in CDCS (Fig. 1D). Notably, functional mutations in at least two of these genes – *BMP4* (bone morphogenetic protein 4) and *GDNF* (glial cell‐derived neurotrophic factor), along with mutations in 13 other DMEGs (for the list of associations, see Table S3) – are known to be associated with a variety of disorders and abnormal developmental phenotypes, including those related to CDCS, such as facial dysmorphism, oral cleft, epicanthus, blindness and vision problems, developmental delays, intellectual disability, and others.

In summary, we provide the first evidence of aberrant DNA methylation in cri‐du‐chat syndrome and its potential involvement in the development of concomitant conditions related to the syndrome. Future research focused on epigenetic mechanisms underlying CDCS may shed additional light on the molecular etiology of this syndrome.

## Conflict of interest

The authors declare no conflict of interest.

## Supporting information


**Table S1.** List of 191 CpGs, which had a significant (Fold Change > 1.2; FDR adjusted *P*‐value < 0.05) difference in the methylation level (*β*‐value) in the participant with Cri‐du‐chat syndrome (CDCS) in comparison to control individuals (CTRL)

**Table S2.** List of 54 genes that contain an Illumina Human Methyalation450 probe localized within a regulatory element (gene promoter, 5′UTR, and the region 1500 nt upstream of the transcription start site, TSS), which had a significant difference in the methylation level (>1.2 fold change at an FDR adjusted *P‐*value < 0.05) in the genome of the participant with Cri‐du‐chat syndrome.

**Table S3.** List of genes differentially methylated in Cri‐du‐chat syndrome, which are known to be associated with a human inherited disease or human phenotypic abnormality. Data on the associations are represented based on the OMIM (Online Mendelian Inheritance in Man; https://omim.org/) and the HPO (The Human Phenotype Ontology; http://human-phenotype-ontology.github.io) data bases.

## References

[1] Bacalini, M. G. , D. Gentilini , A. Boattini , E. Giampieri , C. Pirazzini , C. Giuliani , et al. 2015 Identification of a DNA methylation signature in blood cells from persons with Down Syndrome. Aging (Albany NY) 7:82–93. 25701644 10.18632/aging.100715 PMC4359691

[2] El Hajj, N. , M. Dittrich , J. Böck , T. F. Kraus , I. Nanda , T. Müller , et al. 2016 Epigenetic dysregulation in the developing Down syndrome cortex. Epigenetics 11:563–578. 27245352 10.1080/15592294.2016.1192736 PMC4990229

[3] Jin, S. , Y. K. Lee , Y. C. Lim , Z. Zheng , X. M. Lin , D. P. Ng , et al. 2013 Global DNA hypermethylation in down syndrome placenta. PLoS Genet. 9:e1003515. 23754950 10.1371/journal.pgen.1003515 PMC3675012

[4] McCallie, B. R. , J. C. Parks , A. L. Patton , D. K. Griffin , W. B. Schoolcraft , and M. G. Katz‐Jaffe . 2016 Hypomethylation and genetic instability in monosomy blastocysts may contribute to decreased implantation potential. PLoS ONE 11:e0159507. 27434648 10.1371/journal.pone.0159507 PMC4951028

[5] Sharma, A. , M. A. Jamil , N. Nuesgen , F. Schreiner , L. Priebe , P. Hoffmann , et al. 2015 DNA methylation signature in peripheral blood reveals distinct characteristics of human X chromosome numerical aberrations. Clin. Epigenetics 7:76. 26221191 10.1186/s13148-015-0112-2 PMC4517491

[6] Niebuhr, E. 1978 The Cri du Chat syndrome: epidemiology, cytogenetics, and clinical features. Hum. Genet. 44:227–275. 365706 10.1007/BF00394291

[7] Overhauser, J. , X. Huang , M. Gersh , W. Wilson , J. McMahon , U. Bengtsson , et al. 1994 Molecular and phenotypic mapping of the short arm of chromosome 5: sublocalization of the critical region for the cri‐du‐chat syndrome. Hum. Mol. Genet. 3:242–252. 10.1093/hmg/3.2.247 8004090

[8] Mainardi, P. , C. Perfumo , A. Cali , G. Coucourde , G. Pastore , S. Cavani , et al. 2001 Clinical and molecular characterisation of 80 patients with 5p deletion: genotype‐phenotype correlation. J. Med. Genet. 38:151–158. 11238681 10.1136/jmg.38.3.151 PMC1734829

[9] Medina, M. , R. C. Marinescu , J. Overhauser , and K. S. Kosik . 2000 Hemizygosity of delta‐catenin (CTNND2) is associated with severe mental retardation in cri‐du‐chat syndrome. Genomics 63:157–164. 10673328 10.1006/geno.1999.6090

[10] Simmons, A. D. , A. W. Püschel , J. D. McPherson , J. Overhauser , and M. Lovett . 1998 Molecular cloning and mapping of human Semaphorin F from the Cri‐du‐chat candidate interval. Biochem. Biophys. Res. Comm. 242:685–691. 9464278 10.1006/bbrc.1997.8027

[11] Crider, K. S. , T. P. Yang , R. J. Berry , and L. B. Bailey . 2012 Folate and DNA methylation: a review of molecular mechanisms and the evidence for folate's role. Adv. Nutr. 3:21–38. 22332098 10.3945/an.111.000992 PMC3262611

[12] Orozco, L. D. , M. Morselli , L. Rubbi , W. Guo , J. Go , H. Shi , et al. 2015 Epigenome‐wide association of liver methylation patterns and complex metabolic traits in mice. Cell Metab. 21:905–917. 26039453 10.1016/j.cmet.2015.04.025 PMC4454894

[13] Arakawa, Y. , M. Watanabe , N. Inoue , M. Sarumaru , Y. Hidaka , and Y. Iwatani . 2012 Association of polymorphisms in DNMT1, DNMT3A, DNMT3B, MTHFR and MTRR genes with global DNA methylation levels and prognosis of autoimmune thyroid disease. Clin. Exp. Immunol. 170:194–201. 23039890 10.1111/j.1365-2249.2012.04646.x PMC3482366

[14] Aryee, M. J. , A. E. Jaffe , H. Corrada‐Bravo , C. Ladd‐Acosta , A. P. Feinberg , K. D. Hansen , et al. 2014 Minfi: a flexible and comprehensive Bioconductor package for the analysis of Infinium DNA Methylation microarrays. Bioinformatics 30:1363–1369. 24478339 10.1093/bioinformatics/btu049 PMC4016708

[15] Houseman, E. A. , W. P. Accomando , D. C. Koestler , B. C. Christensen , C. J. Marsit , H. H. Nelson , et al. 2012 DNA methylation arrays as surrogate measures of cell mixture distribution. BMC Bioinformatics 13:86. 22568884 10.1186/1471-2105-13-86 PMC3532182

