# Supplementary material for: DNA methylation alterations in the genome of a toddler with cri‐du‐chat syndrome
Source: Clin Case Rep. 2017 Nov 20;6(1):14–7. doi: 10.1002/ccr3.1274 (PMC5771924; doi:10.1002/ccr3.1274)
Supplement: Supplementary file 1 — Table S1. List of 191 CpGs, which had a significant (Fold Change > 1.2; FDR adjusted P‐value < 0.05) difference in the methylation level (β‐value) in the participant with Cri‐du‐chat syndrome (CDCS) in comparison to control individuals (CTRL) [file CCR3-6-14-s001.docx]

Supplementary Table S1 –

List of 191 CpGs, which had a significant (Fold Change > 1.2; FDR adjusted *p*-value < .05) difference in the methylation level (β-value) in the participant with Cri-du-chat syndrome (CDCS) in comparison to control individuals (CTRL)

| **Target CpG** | **Genome Localization** | | **Relation to Genes and Regulatory regions** | | | |  | **Differential Methylation Statistics** | | | |
| --- | --- | --- | --- | --- | --- | --- | --- | --- | --- | --- | --- |
|  | **Chromosome** | **Position** | **CG Island** | **Regulatory Group** | **Gene Name** | **Gene Group** | **CTRL,**  **Mean β ±SD** | | **Cri du Chat, β-value** | **FoldChange** | **q-val** |
| cg03330818 | chr1 | 1896553 | Island | N/A | KIAA1751 | Body | .7957 | ± .0310 | .3990 | -1.99 | .0465 |
| cg21209485 | chr1 | 2529359 | S_Shore | N/A | MMEL1 | Body | .8018 | ± .0336 | .3611 | -2.22 | .0435 |
| cg15135176 | chr1 | 3044977 | OpenSea | N/A | PRDM16 | Body | .8864 | ± .0171 | .4737 | -1.87 | .0038 |
| cg11404532 | chr1 | 6486842 | S_Shore | Unclassified | ESPN | Body | .8180 | ± .0140 | .4663 | -1.75 | .0033 |
| cg01800148 | chr1 | 9130886 | N_Shore | N/A | SLC2A5 | TSS1500 | .8243 | ± .0236 | .5268 | -1.56 | .0491 |
| cg22877851 | chr1 | 11847167 | OpenSea | N/A | MTHFR | 3'UTR | .8025 | ± .0248 | .4873 | -1.65 | .0474 |
| cg14498674 | chr1 | 41707653 | Island | Promoter | SCMH1 | 5'UTR | .1244 | ± .0100 | .5502 | 4.42 | .0006 |
| cg13566468 | chr1 | 42954129 | OpenSea | N/A |  |  | .8885 | ± .0197 | .4725 | -1.88 | .0066 |
| cg21245975 | chr1 | 85725523 | Island | Promoter | C1orf52 | TSS200 | .1237 | ± .0417 | .6516 | 5.27 | .0481 |
| cg26272228 | chr1 | 95538484 | Island | Promoter | ALG14 | 5'UTR | .0972 | ± .0041 | .0271 | -3.59 | .0162 |
| cg09076339 | chr1 | 226830923 | S_Shore | Unclassified | ITPKB | Body | .1322 | ± .0205 | .4090 | 3.09 | .0390 |
| cg08521967 | chr1 | 228345490 | Island | N/A | GJC2 | Body | .5703 | ± .0283 | .0696 | -8.19 | .0134 |
| cg08158675 | chr1 | 233121790 | N_Shore | N/A | PCNXL2 | Body | .8729 | ± .0204 | .4968 | -1.76 | .0111 |
| cg09119656 | chr2 | 46556344 | OpenSea | N/A | EPAS1 | Body | .8968 | ± .0154 | .6133 | -1.46 | .0111 |
| cg01905046 | chr2 | 85554079 | N_Shore | N/A | TGOLN2 | Body | .1284 | ± .0100 | .2658 | 2.07 | .0365 |
| cg02741327 | chr2 | 135214041 | OpenSea | N/A | TMEM163 | 3'UTR | .8591 | ± .0236 | .1701 | -5.05 | .0019 |
| cg12456269 | chr2 | 142887725 | Island | N/A | LRP1B | Body | .1132 | ± .0038 | .0655 | -1.73 | .0437 |
| cg13483696 | chr3 | 15639924 | N_Shelf | N/A | HACL1 | Body | .9073 | ± .0126 | .6775 | -1.34 | .0119 |
| cg01347786 | chr3 | 29517179 | OpenSea | N/A | RBMS3 | Body | .8793 | ± .0210 | .6068 | -1.45 | .0450 |
| cg23030339 | chr3 | 31703127 | OpenSea | N/A | OSBPL10 | 3'UTR | .9152 | ± .0202 | .5959 | -1.54 | .0213 |
| cg20917322 | chr3 | 48475754 | OpenSea | N/A | CCDC51 | Body | .9386 | ± .0145 | .6452 | -1.45 | .0074 |
| cg17573813 | chr3 | 61237223 | Island | Promoter | FHIT | TSS200 | .0610 | ± .0098 | .9683 | 15.88 | .0000 |
| cg05069228 | chr3 | 61793623 | OpenSea | N/A | PTPRG | Body | .8602 | ± .0205 | .3976 | -2.16 | .0052 |
| cg01228342 | chr3 | 71779135 | OpenSea | Unclassified | EIF4E3 | 5'UTR;TSS1500 | .0487 | ± .0082 | .3215 | 6.60 | .0014 |
| cg12473849 | chr3 | 136882749 | OpenSea | N/A |  |  | .8202 | ± .0420 | .0731 | -11.22 | .0131 |
| cg15295441 | chr3 | 151309377 | OpenSea | N/A |  |  | .9230 | ± .0066 | .5983 | -1.54 | .0004 |
| cg07475940 | chr3 | 170304889 | S_Shore | N/A | SLC7A14 | TSS1500 | .9006 | ± .0217 | .6033 | -1.49 | .0370 |
| cg24131848 | chr3 | 197477845 | S_Shore | Promoter | FYTTD1;KIAA0226 | Body;5'UTR | .1087 | ± .0314 | .6523 | 6.00 | .0149 |
| cg02099267 | chr4 | 963838 | Island | N/A | DGKQ | Body | .9120 | ± .0124 | .5560 | -1.64 | .0019 |
| cg11956442 | chr4 | 39531927 | S_Shelf | N/A |  |  | .0754 | ± .0403 | .7113 | 9.43 | .0213 |
| cg25484904 | chr4 | 48988015 | Island | N/A | CWH43 | TSS1500 | .3122 | ± .0158 | .9405 | 3.01 | .0009 |
| cg20058799 | chr4 | 71706709 | S_Shore | Unclassified | GRSF1 | TSS1500 | .8788 | ± .0135 | .3770 | -2.33 | .0011 |
| cg27581660 | chr4 | 99700350 | OpenSea | N/A |  |  | .8966 | ± .0126 | .6033 | -1.49 | .0045 |
| cg14035247 | chr4 | 127811266 | OpenSea | N/A |  |  | .9058 | ± .0189 | .4961 | -1.83 | .0060 |
| cg18033756 | chr5 | 1660026 | N_Shelf | Unclassified |  |  | .3672 | ± .0105 | .5028 | 1.37 | .0450 |
| cg23517115 | chr5 | 6581849 | N_Shore | N/A | LOC255167 | TSS1500 | .8513 | ± .0226 | .4438 | -1.92 | .0125 |
| cg14590843 | chr5 | 37838290 | Island | N/A | GDNF | 5'UTR | .2630 | ± .0094 | .4552 | 1.73 | .0071 |
| cg01547622 | chr5 | 49962954 | Island | N/A | PARP8 | 1stExon;5'UTR | .0629 | ± .0120 | .3595 | 5.71 | .0036 |
| cg08470036 | chr5 | 64858963 | Island | Promoter | PPWD1;CENPK | TSS200;5'UTR | .0735 | ± .0178 | .5497 | 7.48 | .0026 |
| cg27348779 | chr5 | 92641165 | OpenSea | N/A |  |  | .8024 | ± .0168 | .5221 | -1.54 | .0175 |
| cg10949007 | chr5 | 95159614 | OpenSea | N/A | GLRX | TSS1500 | .1332 | ± .0094 | .4611 | 3.46 | .0012 |
| cg11428066 | chr5 | 134688497 | OpenSea | N/A | H2AFY | Body | .9150 | ± .0111 | .6500 | -1.41 | .0040 |
| cg13614103 | chr5 | 141217652 | OpenSea | N/A |  |  | .9166 | ± .0123 | .7609 | -1.20 | .0485 |
| cg18394648 | chr5 | 141538333 | OpenSea | N/A |  |  | .9427 | ± .0089 | .5604 | -1.68 | .0006 |
| ch.5.3099968F | chr5 | 164258317 | OpenSea | N/A |  |  | .0636 | ± .0033 | .1146 | 1.80 | .0233 |
| cg21082033 | chr5 | 177591349 | N_Shore | Unclassified |  |  | .0511 | ± .0036 | .0971 | 1.90 | .0450 |
| cg23360019 | chr6 | 10073684 | OpenSea | N/A |  |  | .8744 | ± .0176 | .6035 | -1.45 | .0232 |
| cg20209681 | chr6 | 22568625 | N_Shore | N/A | HDGFL1 | TSS1500 | .9027 | ± .0134 | .6412 | -1.41 | .0086 |
| cg17713376 | chr6 | 27525713 | OpenSea | N/A |  |  | .2591 | ± .0155 | .4694 | 1.81 | .0381 |
| cg13584225 | chr6 | 27525731 | OpenSea | N/A |  |  | .1279 | ± .0123 | .3966 | 3.10 | .0058 |
| cg25650661 | chr6 | 29856278 | Island | Promoter | HLA-H | Body | .0885 | ± .0102 | .2421 | 2.74 | .0249 |
| cg09124453 | chr6 | 30074197 | S_Shelf | N/A | TRIM31 | Body | .8784 | ± .0274 | .5122 | -1.71 | .0401 |
| cg12999291 | chr6 | 31123569 | N_Shelf | N/A | CCHCR1 | Body | .8498 | ± .0127 | .5556 | -1.53 | .0045 |
| cg11040238 | chr6 | 31143798 | OpenSea | N/A | PSORS1C3 | Body | .8202 | ± .0207 | .5179 | -1.58 | .0287 |
| cg15415945 | chr6 | 31627678 | N_Shore | N/A | C6orf47 | 1stExon;5'UTR | .9936 | ± .0017 | .7351 | -1.35 | .0000 |
| cg16990996 | chr6 | 31632774 | N_Shore | Promoter | BAT4;CSNK2B | 5'UTR;TSS1500 | .0573 | ± .0008 | .0818 | 1.43 | .0018 |
| cg03317682 | chr6 | 32086091 | OpenSea | N/A | ATF6B | Body | .8854 | ± .0153 | .5418 | -1.63 | .0052 |
| cg21358343 | chr6 | 33263960 | N_Shelf | N/A | RGL2 | Body | .9057 | ± .0114 | .7520 | -1.20 | .0390 |
| cg17256157 | chr6 | 142410100 | Island | N/A | NMBR | TSS200 | .0986 | ± .0060 | .2254 | 2.29 | .0067 |
| cg07895329 | chr6 | 154821545 | OpenSea | N/A | CNKSR3 | Body | .9107 | ± .0143 | .6731 | -1.35 | .0175 |
| cg06699489 | chr6 | 158690902 | OpenSea | N/A |  |  | .8614 | ± .0347 | .3475 | -2.48 | .0273 |
| cg08645225 | chr7 | 779089 | N_Shore | N/A | HEATR2 | Body | .9424 | ± .0113 | .6459 | -1.46 | .0027 |
| cg13945667 | chr7 | 812858 | N_Shore | N/A | HEATR2 | Body | .8540 | ± .0139 | .6000 | -1.42 | .0115 |
| cg12561079 | chr7 | 986139 | S_Shore | N/A | ADAP1 | Body | .9254 | ± .0107 | .5364 | -1.73 | .0011 |
| cg16542356 | chr7 | 1121190 | S_Shore | N/A | C7orf50 | Body | .9130 | ± .0205 | .4871 | -1.87 | .0069 |
| cg07351675 | chr7 | 1578263 | S_Shelf | Promoter | MAFK | 5'UTR | .9369 | ± .0156 | .3682 | -2.54 | .0011 |
| cg09219710 | chr7 | 2629984 | N_Shelf | N/A | IQCE | Body | .9306 | ± .0271 | .5852 | -1.59 | .0474 |
| cg17393140 | chr7 | 2764129 | OpenSea | N/A |  |  | .7868 | ± .0108 | .4936 | -1.59 | .0025 |
| cg24648384 | chr7 | 2802942 | S_Shore | N/A | GNA12 | Body | .8089 | ± .0201 | .4694 | -1.72 | .0168 |
| cg02384857 | chr7 | 27197614 | N_Shore | Unclassified | HOXA7 | TSS1500 | .0562 | ± .0033 | .1521 | 2.71 | .0019 |
| cg08321815 | chr7 | 34494374 | OpenSea | N/A |  |  | .8809 | ± .0234 | .2118 | -4.16 | .0019 |
| cg10000825 | chr7 | 131012516 | Island | Promoter | MKLN1 | TSS200 | .1590 | ± .0058 | .2538 | 1.60 | .0193 |
| cg24022528 | chr7 | 140393175 | N_Shelf | N/A | ADCK2 | Body | .8887 | ± .0175 | .1080 | -8.23 | .0006 |
| cg26054842 | chr7 | 155915712 | Island | N/A |  |  | .8319 | ± .0163 | .5394 | -1.54 | .0127 |
| cg11380909 | chr8 | 6658332 | OpenSea | Unclassified |  |  | .0979 | ± .0040 | .1518 | 1.55 | .0376 |
| cg07781645 | chr8 | 16059680 | OpenSea | N/A |  |  | .8811 | ± .0104 | .5731 | -1.54 | .0019 |
| cg10644890 | chr8 | 41998516 | S_Shore | Promoter |  |  | .0763 | ± .0028 | .1446 | 1.90 | .0036 |
| cg05806180 | chr8 | 70378959 | OpenSea | N/A | SULF1 | 5'UTR;1stExon | .0958 | ± .0066 | .2338 | 2.44 | .0068 |
| cg01470456 | chr8 | 85787158 | OpenSea | N/A | RALYL | Body | .9040 | ± .0140 | .6456 | -1.40 | .0111 |
| cg03067774 | chr8 | 126341151 | OpenSea | N/A | NSMCE2 | Body | .8647 | ± .0214 | .5620 | -1.54 | .0328 |
| cg00776405 | chr8 | 133967862 | OpenSea | N/A | TG | Body | .9147 | ± .0169 | .6673 | -1.37 | .0287 |
| cg10076068 | chr8 | 142139187 | Island | N/A | DENND3 | 5'UTR | .1296 | ± .0361 | .5955 | 4.59 | .0453 |
| cg12103720 | chr8 | 143262572 | OpenSea | N/A |  |  | .8843 | ± .0129 | .6113 | -1.45 | .0067 |
| cg19657945 | chr8 | 144367392 | S_Shore | Unclassified |  |  | .9217 | ± .0140 | .6632 | -1.39 | .0111 |
| ch.9.76081330F | chr9 | 76891510 | OpenSea | N/A |  |  | .0851 | ± .0049 | .1652 | 1.94 | .0194 |
| cg14582550 | chr9 | 97786879 | OpenSea | N/A | C9orf3 | Body | .9132 | ± .0137 | .0423 | -21.58 | .0001 |
| cg21891967 | chr9 | 98075492 | Island | N/A | FANCC | 5'UTR | .5147 | ± .0211 | .2419 | -2.13 | .0450 |
| cg14417873 | chr9 | 136838534 | OpenSea | N/A | VAV2 | Body | .7799 | ± .0277 | .2453 | -3.18 | .0092 |
| cg12128445 | chr10 | 6014983 | N_Shelf | N/A | IL15RA | 5'UTR | .8925 | ± .0261 | .3483 | -2.56 | .0068 |
| cg18372739 | chr10 | 7622106 | S_Shore | N/A | ITIH5 | Body | .7754 | ± .0350 | .3280 | -2.36 | .0468 |
| cg05721515 | chr10 | 8095288 | Island | Promoter | FLJ45983;GATA3 | TSS1500 | .1592 | ± .0169 | .5904 | 3.71 | .0031 |
| cg22660341 | chr10 | 11343618 | OpenSea | N/A | CUGBP2 | Body | .8994 | ± .0111 | .6693 | -1.34 | .0071 |
| cg17843891 | chr10 | 11780853 | N_Shelf | N/A |  |  | .8915 | ± .0337 | .2106 | -4.23 | .0074 |
| cg22710156 | chr10 | 16285821 | OpenSea | N/A |  |  | .8766 | ± .0152 | .5654 | -1.55 | .0071 |
| cg13796975 | chr10 | 21467394 | S_Shelf | N/A |  |  | .8913 | ± .0219 | .5765 | -1.55 | .0310 |
| cg02203761 | chr10 | 27232252 | OpenSea | N/A | NCRNA00202 | TSS1500 | .8783 | ± .0106 | .5674 | -1.55 | .0019 |
| cg25334461 | chr10 | 29875062 | OpenSea | N/A | SVIL | 5'UTR | .8274 | ± .0193 | .4187 | -1.98 | .0066 |
| cg19848924 | chr10 | 34344580 | OpenSea | N/A |  |  | .8533 | ± .0221 | .5100 | -1.67 | .0228 |
| cg13133883 | chr10 | 86004660 | OpenSea | Unclassified | RGR | TSS200 | .5674 | ± .0294 | .9365 | 1.65 | .0496 |
| cg08976451 | chr10 | 92631876 | OpenSea | Promoter | RPP30 | Body | .0664 | ± .0072 | .1676 | 2.52 | .0333 |
| cg18001722 | chr10 | 121146289 | OpenSea | N/A | GRK5 | Body | .9060 | ± .0229 | .5800 | -1.56 | .0317 |
| cg24268035 | chr10 | 126262805 | OpenSea | N/A | LHPP | Body | .9538 | ± .0115 | .7923 | -1.20 | .0333 |
| cg05812143 | chr10 | 127467986 | S_Shelf | N/A |  |  | .7799 | ± .0329 | .3467 | -2.25 | .0426 |
| cg14270494 | chr10 | 133377007 | OpenSea | N/A |  |  | .9004 | ± .0146 | .3771 | -2.39 | .0011 |
| cg03188793 | chr11 | 755658 | OpenSea | N/A | TALDO1 | Body | .9186 | ± .0114 | .5570 | -1.65 | .0017 |
| cg21743649 | chr11 | 3820252 | S_Shore | N/A | PGAP2;NUP98 | TSS1500 | .9061 | ± .0089 | .5235 | -1.73 | .0006 |
| cg25627675 | chr11 | 8707082 | N_Shelf | N/A | RPL27A;SNORA45 | Body | .8568 | ± .0149 | .4062 | -2.11 | .0018 |
| cg11008123 | chr11 | 33097335 | OpenSea | N/A | LOC283267 | TSS1500 | .4585 | ± .0267 | .8344 | 1.82 | .0333 |
| cg02946903 | chr11 | 45742200 | OpenSea | N/A |  |  | .8924 | ± .0198 | .5600 | -1.59 | .0168 |
| cg04217927 | chr11 | 47737105 | Island | Promoter | AGBL2 | TSS1500 | .0583 | ± .0030 | .0986 | 1.69 | .0408 |
| cg01742905 | chr11 | 88071023 | Island | Promoter | CTSC | TSS200 | .0737 | ± .0117 | .9437 | 12.81 | .0001 |
| cg27300950 | chr11 | 117947628 | OpenSea | Unclassified | TMPRSS4 | TSS200 | .8887 | ± .0179 | .5514 | -1.61 | .0104 |
| cg27531470 | chr11 | 133907344 | Island | Unclassified | LOC100128239 | Body | .1176 | ± .0088 | .3693 | 3.14 | .0019 |
| cg19195609 | chr12 | 123464932 | Island | N/A | ARL6IP4 | 1stExon;5'UTR | .0524 | ± .0025 | .0922 | 1.76 | .0195 |
| cg05214130 | chr12 | 123945990 | S_Shelf | N/A | SNRNP35 | 5'UTR | .9079 | ± .0204 | .5149 | -1.76 | .0092 |
| cg09284209 | chr12 | 124585152 | OpenSea | N/A |  |  | .5483 | ± .0224 | .1074 | -5.11 | .0084 |
| cg11132120 | chr12 | 125020161 | N_Shelf | N/A | NCOR2 | TSS200 | .9035 | ± .0160 | .4551 | -1.99 | .0021 |
| cg22697821 | chr12 | 125103252 | OpenSea | N/A |  |  | .8443 | ± .0212 | .5057 | -1.67 | .0205 |
| cg11755803 | chr12 | 129000542 | OpenSea | N/A | TMEM132C | Body | .8942 | ± .0118 | .5367 | -1.67 | .0018 |
| cg08220028 | chr12 | 132834453 | S_Shore | N/A | GALNT9 | Body | .8773 | ± .0162 | .5540 | -1.58 | .0079 |
| cg16151959 | chr13 | 42704154 | OpenSea | N/A | DGKH | Body | .8772 | ± .0171 | .5473 | -1.60 | .0091 |
| cg16596598 | chr13 | 47316618 | OpenSea | N/A | LRCH1 | 3'UTR | .8978 | ± .0171 | .6647 | -1.35 | .0376 |
| cg03272499 | chr13 | 66919912 | OpenSea | N/A | PCDH9 | Body | .8234 | ± .0213 | .4812 | -1.71 | .0201 |
| cg19252199 | chr13 | 110960942 | Island | N/A | COL4A1 | TSS1500 | .0801 | ± .0071 | .2833 | 3.54 | .0019 |
| cg18827503 | chr13 | 112574260 | N_Shore | N/A |  |  | .8960 | ± .0217 | .5293 | -1.69 | .0165 |
| cg19528830 | chr13 | 113685296 | S_Shore | Unclassified | MCF2L | Body | .8554 | ± .0264 | .3142 | -2.72 | .0071 |
| cg15388766 | chr13 | 113688132 | S_Shore | Unclassified | MCF2L | Body | .8964 | ± .0146 | .4486 | -2.00 | .0018 |
| cg00587941 | chr13 | 114741241 | N_Shelf | Unclassified |  |  | .8847 | ± .0161 | .5180 | -1.71 | .0049 |
| cg21418076 | chr14 | 24777878 | N_Shore | N/A | LTB4R2;CIDEB | TSS1500;5'UTR | .9031 | ± .0170 | .5239 | -1.72 | .0052 |
| cg26846476 | chr14 | 24838993 | Island | N/A | NFATC4 | Body | .9294 | ± .0140 | .5699 | -1.63 | .0030 |
| cg09339394 | chr14 | 35008661 | Island | Promoter | EAPP | Body | .0629 | ± .0153 | .8706 | 13.84 | .0003 |
| cg24704332 | chr14 | 54065931 | OpenSea | N/A |  |  | .8538 | ± .0199 | .4481 | -1.91 | .0071 |
| cg04245766 | chr14 | 54421051 | Island | Unclassified | BMP4 | 5'UTR | .1099 | ± .0097 | .4444 | 4.04 | .0012 |
| cg15876198 | chr14 | 95106927 | OpenSea | N/A | SERPINA13 | TSS200 | .8913 | ± .0275 | .5417 | -1.65 | .0474 |
| cg04741516 | chr14 | 99786208 | OpenSea | N/A |  |  | .8912 | ± .0122 | .6931 | -1.29 | .0195 |
| cg24363621 | chr15 | 25430653 | OpenSea | N/A | SNORD115 | TSS200 | .7832 | ± .0215 | .4397 | -1.78 | .0205 |
| cg27384569 | chr15 | 26927633 | OpenSea | N/A | GABRB3 | Body | .8517 | ± .0194 | .5259 | -1.62 | .0168 |
| cg00151124 | chr15 | 39847908 | OpenSea | N/A |  |  | .8280 | ± .0152 | .5948 | -1.39 | .0233 |
| cg01085553 | chr15 | 81225491 | OpenSea | N/A | KIAA1199 | Body | .9160 | ± .0140 | .5565 | -1.65 | .0030 |
| cg22681495 | chr15 | 86058755 | OpenSea | N/A | AKAP13 | Body | .8694 | ± .0260 | .4557 | -1.91 | .0205 |
| cg27018984 | chr15 | 90796557 | S_Shelf | N/A | TTLL13 | Body | .8977 | ± .0183 | .3537 | -2.54 | .0018 |
| cg03478313 | chr15 | 93655850 | S_Shelf | N/A |  |  | .8693 | ± .0103 | .5785 | -1.50 | .0021 |
| cg01733958 | chr16 | 423420 | N_Shore | N/A | TMEM8A | Body | .8641 | ± .0156 | .3458 | -2.50 | .0014 |
| cg01741056 | chr16 | 25075152 | N_Shelf | N/A |  |  | .7807 | ± .0376 | .2536 | -3.08 | .0333 |
| cg07751125 | chr16 | 70557411 | Island | Promoter | COG4;SF3B3 | 1stExon;TSS1500 | .0803 | ± .0049 | .1497 | 1.86 | .0333 |
| cg05363110 | chr16 | 79598137 | OpenSea | N/A |  |  | .8915 | ± .0218 | .5404 | -1.65 | .0200 |
| cg02093708 | chr16 | 87577273 | OpenSea | N/A |  |  | .9132 | ± .0143 | .5650 | -1.62 | .0037 |
| cg18676679 | chr16 | 87984422 | Island | Promoter | BANP | TSS1500 | .1006 | ± .0109 | .5204 | 5.17 | .0010 |
| cg08047086 | chr16 | 89044646 | OpenSea | N/A | CBFA2T3 | TSS1500 | .4636 | ± .0411 | .9950 | 2.15 | .0450 |
| cg15465836 | chr17 | 3371409 | N_Shelf | N/A | SPATA22 | Body | .8452 | ± .0246 | .4110 | -2.06 | .0135 |
| cg20215438 | chr17 | 6541725 | N_Shore | N/A | KIAA0753 | 5'UTR | .8533 | ± .0198 | .5880 | -1.45 | .0397 |
| cg24997359 | chr17 | 21954451 | S_Shore | Unclassified |  |  | .3646 | ± .0123 | .5196 | 1.43 | .0492 |
| cg01979298 | chr17 | 38589733 | OpenSea | Unclassified |  |  | .8996 | ± .0163 | .6957 | -1.29 | .0496 |
| cg05878104 | chr17 | 40932452 | Island | N/A | WNK4 | TSS200 | .2547 | ± .0132 | .5289 | 2.08 | .0069 |
| cg16446617 | chr17 | 43443479 | OpenSea | N/A |  |  | .8649 | ± .0102 | .4992 | -1.73 | .0011 |
| cg21035183 | chr17 | 46872415 | OpenSea | N/A | TTLL6 | TSS1500 | .8235 | ± .0313 | .3422 | -2.41 | .0233 |
| cg21765359 | chr17 | 75246756 | S_Shelf | N/A |  |  | .8705 | ± .0158 | .5838 | -1.49 | .0120 |
| cg16377738 | chr17 | 76141068 | S_Shelf | N/A | C17orf99 | TSS1500 | .8578 | ± .0243 | .3667 | -2.34 | .0074 |
| cg24419602 | chr19 | 5510310 | S_Shelf | N/A |  |  | .9110 | ± .0174 | .6676 | -1.36 | .0333 |
| cg07718631 | chr19 | 17728572 | Island | N/A | UNC13A | Body | .9564 | ± .0118 | .6825 | -1.40 | .0045 |
| cg22996768 | chr19 | 33719749 | S_Shore | N/A |  |  | .0965 | ± .0354 | .6049 | 6.27 | .0310 |
| cg10148932 | chr19 | 39967363 | N_Shelf | N/A |  |  | .8312 | ± .0213 | .1599 | -5.20 | .0017 |
| cg11942206 | chr19 | 43383986 | OpenSea | N/A | PSG1 | TSS200 | .8973 | ± .0183 | .2706 | -3.32 | .0012 |
| cg08436396 | chr19 | 44306947 | S_Shelf | N/A | LYPD5 | TSS1500;5'UTR | .8124 | ± .0206 | .2707 | -3.00 | .0027 |
| cg08228932 | chr19 | 47757612 | N_Shelf | N/A |  |  | .8846 | ± .0223 | .5248 | -1.69 | .0197 |
| cg19746536 | chr19 | 49375674 | Island | Promoter | PPP1R15A | 5'UTR;1stExon | .0897 | ± .0238 | .4583 | 5.11 | .0231 |
| cg01270299 | chr19 | 53099757 | OpenSea | N/A | ZNF137 | TSS200 | .8821 | ± .0315 | .4622 | -1.91 | .0402 |
| cg01421902 | chr19 | 53697534 | S_Shore | N/A | ZNF665 | TSS1500 | .9052 | ± .0210 | .5536 | -1.64 | .0171 |
| cg25979157 | chr19 | 53902687 | S_Shelf | N/A | ZNF765 | Body | .9226 | ± .0237 | .5728 | -1.61 | .0278 |
| cg22866218 | chr19 | 53973920 | S_Shelf | N/A | ZNF813 | 5'UTR | .8785 | ± .0257 | .5439 | -1.62 | .0444 |
| cg20336016 | chr19 | 55038263 | Island | N/A |  |  | .9025 | ± .0164 | .4087 | -2.21 | .0018 |
| cg10747483 | chr19 | 55038290 | Island | N/A |  |  | .9354 | ± .0145 | .6228 | -1.50 | .0060 |
| cg26264314 | chr19 | 56510965 | OpenSea | N/A | NLRP5 | TSS200 | .8810 | ± .0157 | .3981 | -2.21 | .0018 |
| cg17835207 | chr20 | 48524531 | S_Shore | N/A | SPATA2 | Body | .8490 | ± .0198 | .5322 | -1.60 | .0205 |
| cg00272992 | chr20 | 55108228 | OpenSea | N/A | C20orf107 | TSS200 | .9207 | ± .0243 | .5470 | -1.68 | .0233 |
| cg01789728 | chr20 | 57607451 | Island | Promoter | ATP5E | TSS200 | .1220 | ± .0162 | .3324 | 2.73 | .0446 |
| cg11919138 | chr20 | 57607692 | Island | Promoter | ATP5E | TSS1500 | .0628 | ± .0152 | .3653 | 5.82 | .0079 |
| cg14115220 | chr20 | 58533421 | OpenSea | N/A | CDH26 | TSS200 | .8720 | ± .0176 | .3199 | -2.73 | .0017 |
| cg00264578 | chr20 | 59864534 | N_Shore | N/A | CDH4 | Body | .8833 | ± .0113 | .5362 | -1.65 | .0018 |
| cg23096297 | chr20 | 61557774 | Island | Promoter | DIDO1 | 5'UTR | .0764 | ± .0095 | .4388 | 5.75 | .0010 |
| cg01664727 | chr21 | 36258423 | N_Shore | N/A | RUNX1 | Body | .5040 | ± .0302 | .1245 | -4.05 | .0496 |
| cg00994804 | chr21 | 36259383 | Island | N/A | RUNX1 | 1stExon; 5'UTR | .4540 | ± .0242 | .0758 | -5.99 | .0220 |
| cg26790209 | chr21 | 47806332 | S_Shore | N/A | PCNT | Body | .8671 | ± .0142 | .5216 | -1.66 | .0038 |
| cg04234412 | chr22 | 24373322 | Island | Promoter | LOC391322 | Body | .2743 | ± .0432 | .8681 | 3.16 | .0368 |
| cg09033563 | chr22 | 24373618 | Island | N/A | LOC391322 | Body | .1280 | ± .0094 | .5276 | 4.12 | .0006 |
| cg05380919 | chr22 | 24376252 | S_Shelf | N/A | GSTT1 | 3'UTR | .8888 | ± .0154 | .5124 | -1.73 | .0037 |
| cg01744396 | chr22 | 24384245 | Island | Unclassified | GSTT1 | 5'UTR | .0792 | ± .0139 | .3916 | 4.94 | .0052 |
| cg10546252 | chr22 | 24384294 | Island | Unclassified | GSTT1 | TSS200 | .0513 | ± .0042 | .3355 | 6.54 | .0001 |
| cg01500431 | chr22 | 24388327 | S_Shelf | N/A | GSTTP2 | Body | .9189 | ± .0195 | .6125 | -1.50 | .0216 |
| cg20014942 | chr22 | 48977448 | S_Shore | N/A | FAM19A5 | Body | .8522 | ± .0216 | .5372 | -1.59 | .0292 |
| cg21028156 | chrX | 2743660 | N_Shelf | N/A |  |  | .0577 | ± .0175 | .5232 | 9.06 | .0027 |
