# Supplementary material for: DNA methylation alterations in the genome of a toddler with cri‐du‐chat syndrome
Source: Clin Case Rep. 2017 Nov 20;6(1):14–7. doi: 10.1002/ccr3.1274 (PMC5771924; doi:10.1002/ccr3.1274)
Supplement: Supplementary file 2 — Table S2. List of 54 genes that contain an Illumina Human Methyalation450 probe localized within a regulatory element (gene promoter, 5′UTR, and the region 1500 nt upstream of the transcription start site, TSS), which had a significant difference in the methylation level (>1.2 fold change at an FDR adjusted P‐value < 0.05) in the genome of the participant with Cri‐du‐chat syndrome. [file CCR3-6-14-s002.docx]

Supplementary Table S2 –

List of 54 genes that contain an Illumina Human Methyalation450 probe localized within a regulatory element (gene promoter, 5’UTR, and the region 1500 nt upstream of the transcription start site, TSS), which had a significant difference in the methylation level (> 1.2 fold change at an FDR adjusted *p-*value < .05) in the genome of the participant with Cri-du-chat syndrome.

| **Gene Symbol** | **Gene Name** | **Regulatory Region** | **DiffME in CDCS** |
| --- | --- | --- | --- |
| AGBL2 | ATP/GTP binding protein like 2 | Promoter; TSS1500 | up |
| ALG14 | ALG14, UDP-N-acetylglucosaminyltransferase subunit | Promoter; 5'UTR | down |
| ARL6IP4 | ADP ribosylation factor like GTPase 6 interacting protein 4 | 5'UTR | up |
| ATP5E | ATP synthase, H+ transporting, mitochondrial F1 complex, epsilon subunit | Promoter; TSS1500 | up |
| BANP | BTG3 associated nuclear protein | Promoter; TSS1500 | up |
| BMP4 | bone morphogenetic protein 4 | 5'UTR | up |
| CBFA2T3 | CBFA2/RUNX1 translocation partner 3 | TSS1500 | up |
| COL4A1 | collagen type IV alpha 1 chain | TSS1500 | up |
| CTSC | cathepsin C | Promoter; TSS200 | up |
| CWH43 | cell wall biogenesis 43 C-terminal homolog | TSS1500 | up |
| DENND3 | DENN domain containing 3 | 5'UTR | up |
| DIDO1 | death inducer-obliterator 1 | Promoter; 5'UTR | up |
| EAPP | E2F associated phosphoprotein | Promoter | up |
| EIF4E3 | eukaryotic translation initiation factor 4E family member 3 | 5'UTR | up |
| FANCC | Fanconi anemia complementation group C | 5'UTR | down |
| FHIT | fragile histidine triad | Promoter; TSS200 | up |
| GATA3 | GATA binding protein 3 | Promoter; TSS1500 | up |
| GDNF | glial cell derived neurotrophic factor | 5'UTR | up |
| GLRX | glutaredoxin | TSS1500 | up |
| GPANK1 | G-patch domain and ankyrin repeats 1 | Promoter; 5'UTR | up |
| GRSF1 | G-rich RNA sequence binding factor 1 | TSS1500 | down |
| GSTT1 | glutathione S-transferase theta 1 | 5'UTR | up |
| HDGFL1 | hepatoma derived growth factor-like 1 | TSS1500 | down |
| HLA-H | major histocompatibility complex, class I, H | Promoter | up |
| HOXA7 | homeobox A7 | TSS1500 | up |
| IL15RA | interleukin 15 receptor subunit alpha | 5'UTR | down |
| KIAA0753 | OFD1 And FOPNL interacting protein | 5'UTR | down |
| LOC391322 | D-dopachrome tautomerase-like | Promoter | up |
| LYPD5 | LY6/PLAUR domain containing 5 | 5'UTR | down |
| MAFK | MAF bZIP transcription factor K | Promoter; 5'UTR | down |
| MKLN1 | muskelin 1 | Promoter; TSS200 | up |
| NCOR2 | nuclear receptor corepressor 2 | TSS200 | down |
| NLRP5 | NLR family pyrin domain containing 5 | TSS200 | down |
| NMBR | neuromedin B receptor | TSS200 | up |
| PARP8 | poly(ADP-ribose) polymerase family member 8 | 5'UTR | up |
| PGAP2 | post-GPI attachment to proteins 2 | TSS1500 | down |
| PPP1R15A | protein phosphatase 1 regulatory subunit 15A | Promoter; 5'UTR | up |
| PPWD1 | peptidylprolyl isomerase domain and WD repeat containing 1 | Promoter; 5'UTR | up |
| PSG1 | pregnancy specific beta-1-glycoprotein 1 | TSS200 | down |
| RGR | retinal G protein coupled receptor | TSS200 | up |
| RPP30 | ribonuclease P/MRP subunit p30 | Promoter | up |
| RUBCN | RUN and cysteine rich domain containing beclin 1 interacting protein | Promoter; 5'UTR | up |
| RUNX1 | runt related transcription factor 1 | 5'UTR | down |
| SCMH1 | sex comb on midleg homolog 1 | Promoter; 5'UTR | up |
| SLC2A5 | solute carrier family 2 member 5 | TSS1500 | down |
| SLC7A14 | solute carrier family 7 member 14 | TSS1500 | down |
| SNRNP35 | small nuclear ribonucleoprotein U11/U12 subunit 35 | 5'UTR | down |
| SULF1 | sulfatase 1 | 5'UTR | up |
| SVIL | supervillin | 5'UTR | down |
| TMPRSS4 | transmembrane protease, serine 4 | TSS200 | down |
| TTLL6 | tubulin tyrosine ligase like 6 | TSS1500 | down |
| WNK4 | WNK lysine deficient protein kinase 4 | TSS200 | up |
| ZNF665 | zinc finger protein 665 | TSS1500 | down |
| ZNF813 | zinc finger protein 813 | 5'UTR | down |
