# Supplementary material for: DNA methylation alterations in the genome of a toddler with cri‐du‐chat syndrome
Source: Clin Case Rep. 2017 Nov 20;6(1):14–7. doi: 10.1002/ccr3.1274 (PMC5771924; doi:10.1002/ccr3.1274)
Supplement: Supplementary file 3 — Table S3. List of genes differentially methylated in Cri‐du‐chat syndrome, which are known to be associated with a human inherited disease or human phenotypic abnormality. Data on the associations are represented based on the OMIM (Online Mendelian Inheritance in Man; https://omim.org/) and the HPO (The Human Phenotype Ontology; http://human-phenotype-ontology.github.io) data bases. [file CCR3-6-14-s003.docx]

Supplementary Table S3 –

List of genes differentially methylated in Cri-du-chat syndrome, which are known to be associated with a human inherited disease or human phenotypic abnormality. Data on the associations are represented based on the OMIM (Online Mendelian Inheritance in Man; <https://omim.org/> ) and the HPO (The Human Phenotype Ontology; <http://human-phenotype-ontology.github.io>) data bases.

| **Gene Symbol** | **OMIM Disease** | **HP Phenotype** |
| --- | --- | --- |
| ALG14 | 616227:Myasthenic syndrome, congenital | 0003403:EMG,ecremental response of compound muscle action potential to repetitive nerve stimulation;  0000508:Ptosis;  002828:Multiple joint contractures;  0003473:Fatigable weakness |
| ATP5E | 614053:Mitochondrial complex V deficiency, nuclear type 3 | 0009830:Peripheral neuropathy;  0001639:Hypertrophic cardiomyopathy;  0003128:Lactic acidosis;  0001249:Intellectual disability |
| BMP4 | 600625:Orofacial cleft 11; 607932:Microphthalmia, syndromic 6 | 0003319:Abnormality of the cervical spine;  0006829:Severe muscular hypotonia;  0000324:Facial asymmetry;  0000202:Oral cleft;  0000286:Epicanthus;  0000568:Microphthalmia;  0001263:Global developmental delay;  0002188:Delayed CNS myelination;  0000618:Blindness;  Others-95 HP Terms |
| COL4A1 | 175780:Porencephaly 1;  180000:Retinal arteries;  607595:Brain small vessel disease with or without ocular anomalies;  611773:Angiopathy, hereditary, with nephropathy, aneurysms, and muscle cramps;  614519:Hemorrhage, intracerebral | 0001263:Global developmental delay;  0001256:Intellectual disability;  0007676:Hypoplasia of the iris;  0000112:Nephropathy;  0000519:Congenital cataract;  0002451:Limb dystonia;  0000577:Exotropia;  Others - 56 HP Terms |
| CTSC | 170650:Periodontitis 1, juvenile;  245000:Papillon-Lefevre syndrome;  245010:Haim-Munk syndrome | 0008404:Nail dystrophy;  0006480:Premature loss of teeth;  0002205:Recurrent respiratory infections;  0002514:Cerebral calcification;  0000951:Abnormality of the skin;  Others - 35 HP Terms |
| FANCC | 227645:Fanconi anemia, complementation group C | 0006265:Aplasia/Hypoplasia of fingers;  0000324:Facial asymmetry;  0006824:Cranial nerve paralysis;  0000286:Epicanthus;  0001824:Weight loss;  0001263:Global developmental delay;  0008572:External ear malformation;  0000508:Ptosis  Others - 112 HP Terms |
| GATA3 | 146255:Hypoparathyroidism, sensorineural deafness, and renal dysplasia | 0000407:Sensorineural hearing impairment;  0012622:Chronic kidney disease;  0000100:Nephrotic syndrome;  0000110:Renal dysplasia;  0003762:Uterus didelphys;  0000829:Hypoparathyroidism |
| GDNF | 171300:Pheochromocytoma;  209880:Central hypoventilation syndrome  613711:Hirschsprung disease, 3 | 0001626:Abnormality of the cardiovascular system;  0001824:Weight loss;  0000369:Low-set ears;  0001252:Muscular hypotonia;  0001249:Intellectual disability;  0000153:Abnormality of the mouth;  Others - 31 HP Terms |
| KIAA0753 | 617127:Orofaciodigital syndrome XV |  |
| PGAP2 | 614207:Hyperphosphatasia with mental retardation syndrome 3 | 0010864:Severe Intellectual disability;  0002059:Cerebral atrophy;  0000252:Microcephaly;  0002905:Hyperphosphatemia;  0001252:Muscular hypotonia;  0001263:Global developmental delay |
| RGR | 613769:Retinitis pigmentosa 44 | 0000505:Visual impairment;  0000648:Optic atrophy;  0000405:Conductive hearing impairment; 0001347:Hyperreflexia;  0001249:Intellectual disability;  0000135:Hypogonadism;  Others – 30 HP Terms |
| RUBCN | 615705:Spinocerebellar ataxia, autosomal recessive 15 | 0001270:Motor delay;  0001249:Intellectual disability;  0001251:Ataxia;  0001272:Cerebellar atrophy |
| RUNX1 | 601399:Platelet disorder, familial, with associated myeloid malignancy;  601626:Leukemia, acute myeloid | 0004845:Acute monocytic leukemia;  0002665:Lymphoma;  0002863:Myelodysplasia;  0003006:Neuroblastoma;  0000421:Epistaxis;  0003540:Impaired platelet aggregation;  Others - 10 HP Terms |
| SLC7A14 | 615725:Retinitis pigmentosa 68 | 0000648:Optic atrophy;  0000618:Blindness;  0000405:Conductive hearing impairment;  0001249:Intellectual disability;  0001347:Hyperreflexia |
| WNK4 | 614491:Pseudohypoaldosteronism, type IIB | 0000822:Hypertension;  0011423:Hyperchloremia;  0008242:Pseudohypoaldosteronism;  0002153:Hyperkalemia |
